# Nanoporous Gold Films Prepared by a Combination of Sputtering and Dealloying for Trace Detection of Benzo[a]pyrene Based on Surface Plasmon Resonance Spectroscopy

**DOI:** 10.3390/s17061255

**Published:** 2017-06-01

**Authors:** Li Wang, Xiu-Mei Wan, Ran Gao, Dan-Feng Lu, Zhi-Mei Qi

**Affiliations:** 1 State Key Laboratory of Transducer Technology, Institute of Electronics, Chinese Academy of Sciences, Beijing 100190, China; wangli1303@163.com (L.W.); wanxiumei1964@163.com (X.-M.W.); rgao@mail.ie.ac.cn (R.G.); 2 School of Electronic, Electrical and Communication Engineering, University of Chinese Academy of Sciences, Beijing 100049, China

**Keywords:** nanoporous gold film, surface plasmon resonance, benzo[a]pyrene, high sensitivity

## Abstract

A wavelength-interrogated surface plasmon resonance (SPR) sensor based on a nanoporous gold (NPG) film has been fabricated for the sensitive detection of trace quantities of benzo[a]pyrene (BaP) in water. The large-area uniform NPG film was prepared by a two-step process that includes sputtering deposition of a 60-nm-thick AuAg alloy film on a glass substrate and chemical dealloying of the alloy film in nitric acid. For SPR sensor applications, the NPG film plays the dual roles of analyte enrichment and supporting surface plasmon waves, which leads to sensitivity enhancement. In this work, the as-prepared NPG film was first modified with 1-dodecanethiol molecules to make the film hydrophobic so as to improve BaP enrichment from water via hydrophobic interactions. The SPR sensor with the hydrophobic NPG film enables one to detect BaP at concentrations as low as 1 nmol·L^−1^. In response to this concentration of BaP the sensor produced a resonance-wavelength shift of Δλ_R_ = 2.22 nm. After the NPG film was functionalized with mouse monoclonal IgG1 that is the antibody against BaP, the sensor’s sensitivity was further improved and the BaP detection limit decreased further down to 5 pmol·L^−1^ (the corresponding Δλ_R_ = 1.77 nm). In contrast, the conventional SPR sensor with an antibody-functionalized dense gold film can give a response of merely Δλ_R_ = 0.9 nm for 100 pmol·L^−1^ BaP.

## 1. Introduction

Polycyclic aromatic hydrocarbons (PAHs) are a group of persistent organic pollutants (POPs) generally containing at least two conjoined aromatic rings, and they are highly carcinogenic, teratogenic and mutagenic [1,2,3]. Benzo[a]pyrene (BaP) is one of the most toxic members of the PAH family, and can cause skin cancer and lung cancer and gastrointestinal cancer. BaP is usually generated from incomplete burning of organic materials and is widely present in air and water, and it is one of the main targets of environmental pollution monitoring [4,5,6]. The environmental protection agency (EPA) of America regulated that the Maximum Contaminant Level (MCL) of BaP in drinking water is 0.0002 mg/L (i.e., 0.2 ppb) [7]. The Chinese government has set national standards for drinking water quality in 1985 that show the MCL of 0.01 μg/L (i.e., 10 ppt) for BaP in drinking water [8]. Low-concentration BaP in air and water is colorless and odorless and thus cannot be easily detected using a conventional sensor. For accurate detection of BaP at ppb and ppt levels in air and water, traditional laboratory analysis techniques have been used, including high performance liquid chromatography (HPLC) [9], gas chromatography-mass spectrometry (GC-MS) [10], surface enhanced Raman spectroscopy (SERS) [11] and fluorescence spectroscopy [12]. These techniques require expensive benchtop instruments and thus are not suitable for on-site analysis. For real-time on-site and ultratrace detection of BaP in air and water, much effort needs to be focused on development of novel high-performance sensors.

BaP is a small molecule with a molecular weight of 252 Da, while its refractive index is as high as 1.887 [13]. These characteristics of BaP give us the idea that compared with a gravimetric sensor such as quartz crystal microbalance (QCM), a refractometric sensor would be more suitable for detection of trace BaP in air and water. It is well known that surface plasmon resonance (SPR) sensors are refractive-index-sensitive devices with label-free detection capability [14,15]. However, a conventional SPR sensor with a dense gold film enables detection of large biomolecules, and its sensitivity is not sufficient for direct detection of small molecules [16]. This is so because the dense gold film allows for formation of only a monolayer of analyte molecules that limits the depth of interaction between the plasmon field and analyte molecules to the monolayer thickness [17]. To overcome this difficulty, a layer of porous dielectric film was immobilized onto the SPR sensor chip. Porous dielectric films can adsorb a large amount of analyte molecules and thus enable to greatly enhance the sensitivity of SPR sensor [18,19,20], which meanwhile make the sensor chip fabrication complicated. An alternative approach is use of nanoporous gold (NPG) films as a SPR sensor [21,22,23]. For SPR sensor application, the NPG film plays the double roles of analyte enrichment and supporting surface plasmon wave, which can result in sensitivity enhancement in the meantime keeping the simple structure of SPR sensors. Compared with common porous dielectric films, NPG film can be easily functionalized using the well-established thiol-gold based surface chemistry [24]. In this work, the uniform NPG films were fabricated by the combination of sputtering deposition and chemical dealloying, and they were used as a wavelength-interrogated SPR sensor for label-free detection of trace BaP in water. After functionalization of the NPG film with the antibody molecules, the SPR sensor enabled the BaP detection at ppt level. 

## 2. Experiments

### 2.1. Reagents and Materials

Standard benzo[a]pyrene/methanol sample solution (5.26 µg/mL, HPLC) obtained from the National Institute of Metrology (Beijing, China) was used to prepare different concentrations of BaP sample aqueous solution; 1-dodecanethiol (C_12_H_25_SH, 98 wt %), 3-mercaptopropionic acid (C_3_H_6_O_2_S, 98 wt %), N-hydroxysuccinimide (NHS, 98 wt %) and N-(3-dimethylaminopropy)-N’-ethyl-carbodiimide hydrochloride (EDC, 98 wt %) were purchased from Aladdin Industrial Corporation (Shanghai, China), and they were used during the modification process of the sensor chip; Mouse monoclonal IgG1 (BaP antibodies, 100 μg·mL^−1^) used for immobilization on the surface of the sensor chip was provided by Santa Cruz Biotechnology (Shanghai, China); Milli-Q deionized water and anhydrous ethanol were used for analyte preparation and acetone applied to clean slide glass substrates were bought from Matsunami Glass Ind., Ltd. (Osaka, Japan) All the reagents above are analytical grade and used as received.

### 2.2. Experimental Setup of the SPR Sensor Platform

**Figure 1:**
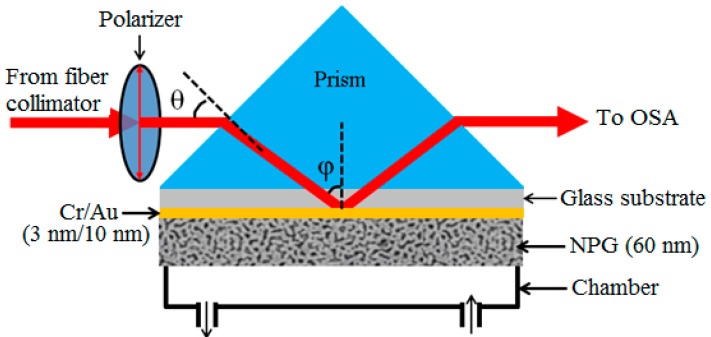
Schematic diagram of the NPG-film-based wavelength-interrogated SPR sensor platform used in this work.

Figure 1 schematically shows the NPG-film based SPR sensor that operates in the wavelength interrogation mode. The sensor was constructed using a tungsten-halogen lamp, a glass prism (45°/45°/90°), an optical spectrum analyzer (OSA), a sample chamber and a NPG-film-based SPR chip. The SPR chip is tightly sandwiched between the prism and the chamber and the assembly is then mounted on a rotating stage. Broadband light from a tungsten-halogen lamp passes through a fiber collimator and a linear polarizer to produce a TM-polarized collimated light beam, and the beam is launched in the prism to undergo the total internal reflection at the glass/NPG interface for resonant coupling of the input power at a specific wavelength to the surface plasmon wave. The reflected light beam is guided with a quartz fiber into the OSA for determination of the resonance wavelength (λ_R_). The initial λ_R_ is adjusted by rotating the stage to change the angle θ between the fixed incident beam and the prism-surface normal. The OSA used in this work is a charge-coupled-device (CCD) spectrometer whose time resolution is 1 ms. A peristaltic pump is used for injection (or removal) of the solution sample into (or out of) the chamber.

### 2.3. Fabrication of the NPG Film 

The NPG films were prepared by a two-step process consisting of sputtering deposition and chemical dealloying. In the first step, a 3-nm chromium layer and a 10-nm gold layer and a 60-nm AuAg alloy film were successively sputtered on the cleaned glass substrates, and this step allows for nanometer-scale controlling of the film thickness. In the second step, the film-covered glass substrate was immersed in nitric acid (68 wt % HNO_3_) at room temperature (about 20 °C) to dissolve the Ag component from the alloy film. The silver dissolution process is accompanied with the self-assembly of gold atoms on the glass substrate that lead to the thermally stable nanoporous structure. In this work the immersion time is controlled to be 4 min. After the substrate was removed out of the nitric acid and thoroughly rinsed with deionized water and completely dried with nitrogen gas, the resulting film is uniform, with a brown color. It is worth noting that the chromium layer is extremely useful for strengthening the adhesion of its upper layer to the glass substrate and the gold layer is employed to protect the chromium layer from dissolution in nitric acid during the process of chemical dealloying. The Cr/Au double-layer structure allows the top NPG layer to have good long-term stability. The energy dispersive X-ray spectroscopy (EDX) results indicate that the as-prepared AuAg alloy film has a weight ratio of Au:Ag ≈ 1:1, almost equal to that for the sputtering alloy target used. 

## 3. Results and Discussions

### 3.1. SEM Characterization of the NPG Film

**Figure 2:**
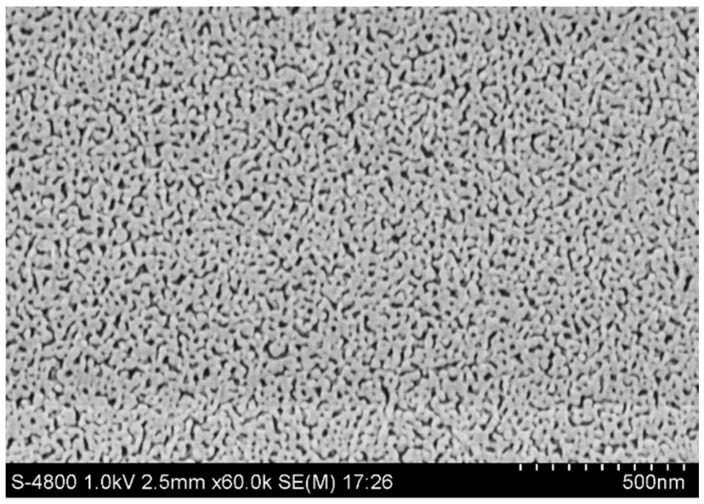
Scanning electron microscope (SEM) image of the as-prepared NPG film.

The surface morphology of the as-prepared NPG film was investigated using a scanning electron microscope (SEM). The SEM image in Figure 2 reveals that the NPG film consists of an interconnected gold framework in which the nanoscale irregular open pores are randomly and evenly embedded. Such morphology offers the NPG film huge internal surface area that is easily available for external molecules, enabling to result in high sensitivity and rapid response of the SPR sensor. Figure 2 also indicates that the NPG film can be treated as an optically homogeneous material in the wavelength range from visible to near infrared. NPG films possess both propagating and localized surface plasmon resonance (SPR) effects that are responsible for widespread applications as chemical and biological SPR sensors. This work is focused on the chemical sensor application of NPG films based on the surface plasmon wave propagating at the NPG/dielectric interface.

### 3.2. Surface Functionalization of the NPG Film 

It is mentioned above that for SPR sensor application, the NPG film plays the two roles of analyte enrichment and supporting surface plasmon wave. It is the dual-role characteristic of the NPG film that offers the resulting SPR sensor high sensitivity as compared with a conventional SPR device. However, BaP molecules without having functional groups shows poor affinity to noble metals, it is difficult to make the effective enrichment of BaP molecules in the NPG film. To overcome this difficulty, the as-prepared NPG film was modified with 1-dodecanethiol based on the thiol-gold surface chemistry. The modified NPG film becomes hydrophobic, enabling to adsorb BaP molecules from water via the hydrophobic interaction. 

**Figure 3:**
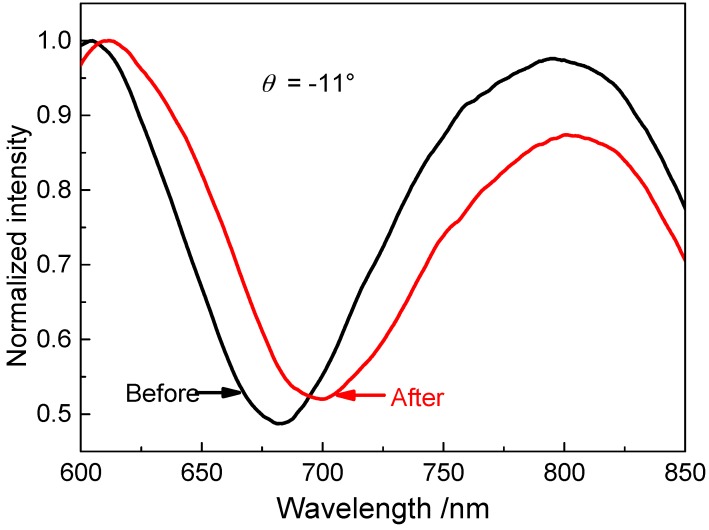
SPR spectra measured at θ = −11° with the NPG film exposed to air before and after modification with 1-dodecanethiol.

The modification process was performed by immersing the NPG film coated glass substrate in an ethanolic solution of 40 mmol·L^−1^ 1-dodecanethiol for 24 h at room temperature. The substrate, after taken out of the solution, was completely rinsed with ethanol and dried with nitrogen gas. Figure 3 displays SPR spectra measured at θ = −11° with the NPG film in air before and after surface modification, from which the resonance wavelength is determined to be λ_R_ = 681.86 nm before modification and λ_R_ = 699.21 nm after modification. The resonance-wavelength shift is Δλ_R_ = 17.35 nm. Such a large shift in λ_R_ gives an indication that the gold-thiol (Au-S) bonds were successfully formed on the outer and inner surfaces of the NPG film.

Hydrophobic interaction based enrichment of BaP molecules in the NPG film can improve the sensor’s sensitivity rather than its selectivity. To improve the sensor’s selectivity for BaP, the NPG film was functionalized with the BaP-against antibody. This functionalization was fulfilled in three steps. In first step, about 40 mL ethanolic solution of 10 mmol·L^−1^ 3-mercaptopropionic acid was pumped into the chamber to form a thiol-gold bonded monolayer on the NPG surface. The monolayer is terminated with carboxyl groups. After the alcoholic solution was held in the chamber at room temperature for about 12 h, the NPG film was rinsed with enough ethanol and deionized water to make sure that excess 3-mercaptopropionic acid molecules were totally washed off.

In the second step, 20 mL of NHS/EDC mixture in water (prepared in accordance with n (EDC)/n (NHS) = 4:1, NHS 10 mg·L^−1^) was injected in the chamber to activate the carboxyl group of the monolayer at room temperature (i.e., amidation of carboxyl groups). After approximately 20 min, the solution was removed from the chamber and the NPG film was rinsed with sufficient water. In the final step, 10 mL antibody solution (in which V (IgG1))/V (PBS) = 0.0031) was kept in the chamber for about 4–5 h at room temperature, making the antibody molecules bond to the NPG film with use of the carboxyl group activated monolayer as linkage. After each operation step, the NPG film was dried in air, then SPR spectrum was measured at θ = −11°. 

**Figure 4:**
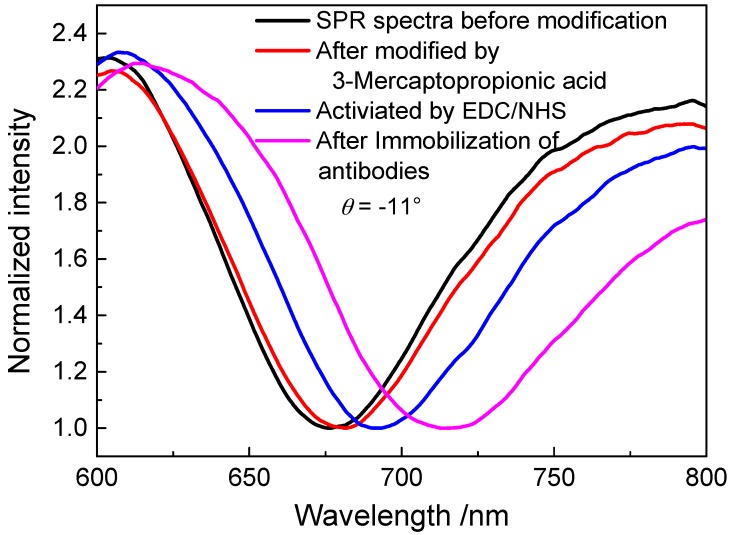
SPR spectra measured at θ = −11° with the dried NPG film before the monolayer formation (black), after the monolayer formation (red), after activation of carboxyl groups (blue), and after antibody immobilization (pink).

Figure 4 displays four SPR spectra and the spectrum in black corresponding to the NPG film before starting the first step. The 1st-step operation makes the resonance wavelength shift from λ_R_ = 676.07 nm to λ_R_ = 680.52 nm, and the redshift is Δλ_R_ = 4.45 nm. The 2nd-step makes the resonance wavelength shift from λ_R_ = 680.52 nm to λ_R_ = 691.21 nm, and the redshift is Δλ_R_ = 10.69 nm; The 3rd-step leads to a redshift of Δλ_R_ = 23.53 nm.

### 3.3. Detection of BaP by the SPR Sensor with the Hydrophobic NPG Film

**Figure 5:**
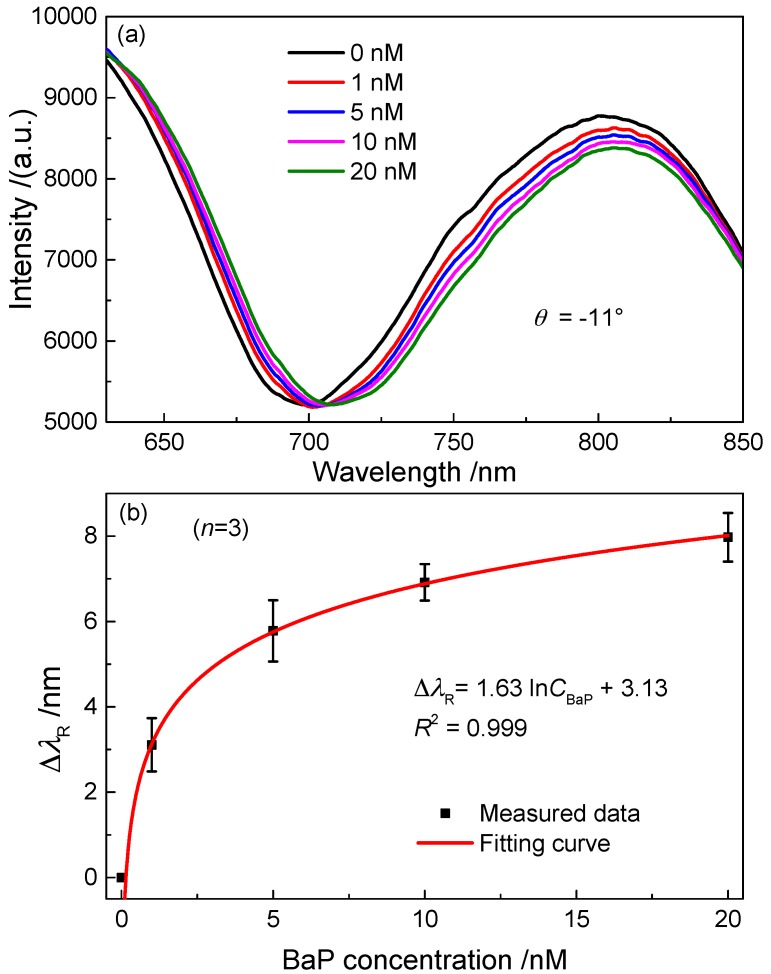
(**a**) SPR spectra measured at θ = −11° with the hydrophobic NPG film in air after adsorption BaP molecules from a series of aqueous solutions with different BaP concentrations; (**b**) the resonance-wavelength shift as a function of the BaP concentration.

The NPG films modified with 1-dodecanethiol were used as a SPR sensor for detection of BaP in water. A series of different concentrations of BaP aqueous solutions, such as 20, 10, 5 and 1 nmol·L^−1^, were prepared by stepwise dilution of standard benzo[a]pyrene/methanol sample solution with deionized water. The SPR spectrum was first measured at θ = −11° with the hydrophobic NPG film in air. Then the four solutions were successively injected into the chamber according to the order from lower concentration to higher concentration. Each solution was kept in the chamber for 20 min, within which the BaP adsorption almost reached equilibrium demonstrated in the experimental. Then the solutions were removed out of the chamber. Figure 5a shows the SPR spectra measured when the sensor chip was sufficiently dried. The resonant wavelength gradually moves to longer wavelength with increasing the BaP concentration.

Figure 5b displays the BaP concentration dependence of Δλ_R_. The error bars were obtained by calculating the standard deviations of three measured Δλ_R_ for each concentration. The experimental results indicate that the BaP with a concentration as low as 1 nmol·L^−1^ could be detected using the hydrophobic NPG film. In response to this concentration of BaP the SPR sensor produced a resonance-wavelength shift of Δλ_R_ = 2.22 nm.

### 3.4. Detection of BaP by the SPR Sensor with the NPG Film Functionalized with the BaP-against Antibody

The response of the SPR sensor with the antibody-functionalized NPG film to BaP molecules was investigated. To do this, a series of BaP aqueous solutions with different concentrations ranging from 5 pmol·L^−1^ to 300 pmol·L^−1^ were prepared as the samples. Firstly, the SPR spectrum was measured at θ = −11° with the modified NPG film in air. Then the BaP solution with the concentration of 5 pmol·L^−1^ was injected into the chamber. After approximate 20 min, the solution was pumped out from the chamber and the NPG film was rinsed with enough deionized water. The SPR spectrum was recorded when the NPG film was completely dried in air. The response spectra for BaP solution with higher concentration were obtained in the same way as mentioned above.

**Figure 6:**
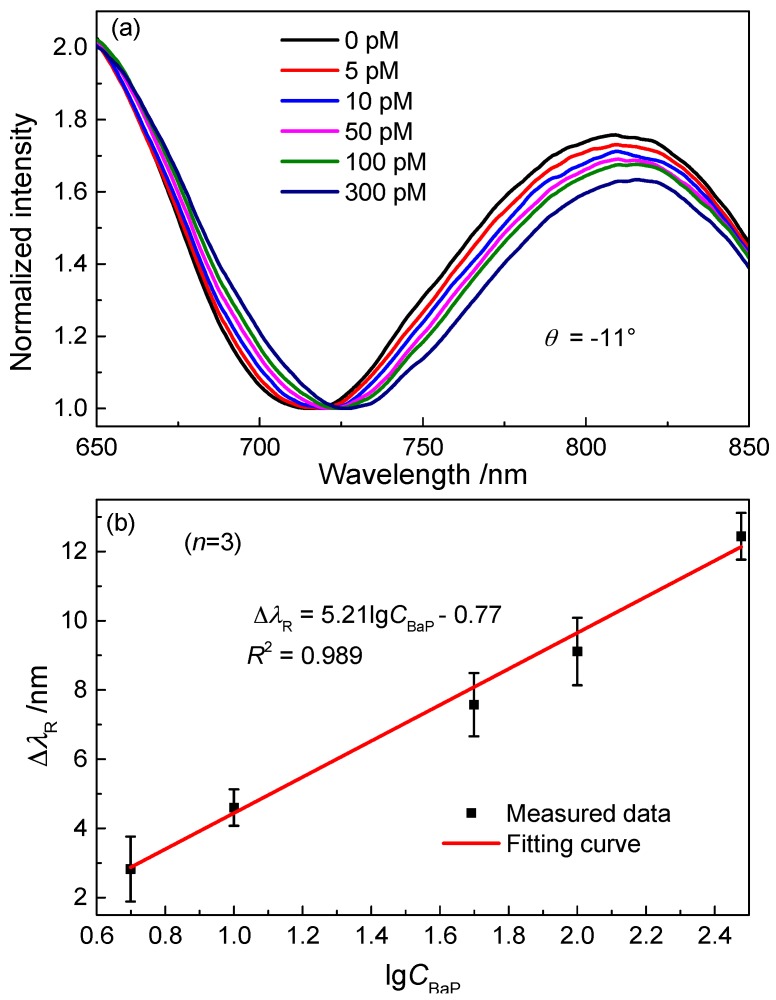
(**a**) SPR spectra measured at θ = −11° with the antibody-functionalized NPG film in air after immunoreaction with BaP molecules in a series of aqueous solutions with different BaP concentrations; (**b**) the resonance-wavelength shift versus the log of BaP concentration.

Figure 6a shows the normalized SPR spectra after immunoreaction with a series of aqueous solutions with different BaP concentrations. The findings indicate that the sensitivity is further improved and the SPR sensor can detect the BaP solution with the concentration of 5 pmol·L^−1^. The corresponding resonance-wavelength shift is Δλ_R_ = 1.77 nm. Figure 6b shows the relationship between Δλ_R_ and the logarithm of BaP concentration. The analytical performance of the measured SPR sensor was comparable with other sensors in earlier reports. A Fe_3_O_4_@Au SERS substrate has been applied to detect the 16 EPA priority PAHs, the detection limit for BaP is 5 nmol·L^−1^ [25]. The BaP detection limit of a SPR immunosensor by employing a self-assembled mixed monolayer presenting BaP head groups was 50 ppt (about 0.2 nmol·L^−1^) [26]. Based on a dual amplification strategy of PAMAM dendrimer and amino-modified methylene blue/SiO_2_ core-shell nanoparticles, a detection limit of 6 pg/mL (about 23.8 pmol·L^−1^) was obtained for the electrochemical immunosensor [27]. An electro-switchable biosurface could detect 10 ng/L (about 40 pmol·L^−1^) BaP by an indirect immunoassay format [28]. The main types of sensors for BaP detection as well as their detection limits are included in Table 1. Such a low detection limit in this work could be contributed to the dual-role characteristic of the NPG film mentioned above. 

### 3.5. Comparison of Sensitivity between the NPG-Film-Based SPR Sensor and That with Dense Gold Film 

**Figure 7:**
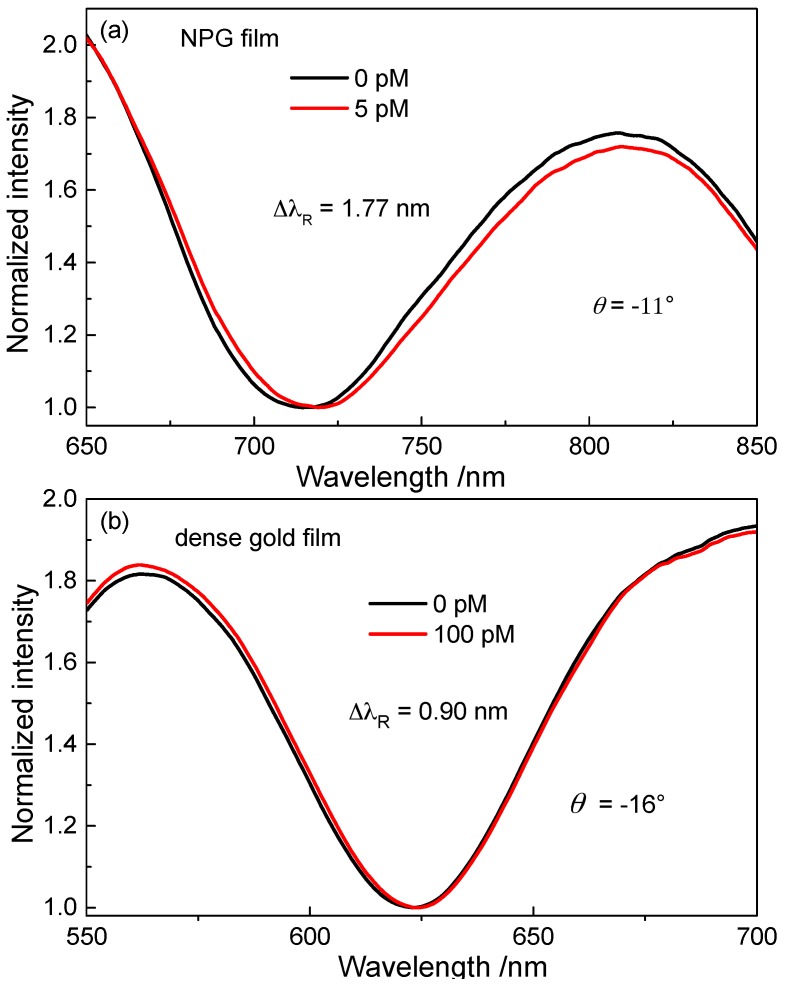
(**a**) SPR spectra measured with the NPG film in air after adsorption of BaP molecules from the aqueous solution with concentration of 5 pmol·L^−1^; (**b**) SPR spectra measured with the dense gold film in air after adsorption of BaP molecules from the aqueous solution with concentration of 100 pmol·L^−1^.

To demonstrate the high sensitivity of the NPG-SPR sensors, the response of a conventional SPR sensor with dense gold film to BaP molecules was investigated. The gold film was modified with BaP-against antibody by the same method as used for the NPG film. Figure 7a,b display the measured SPR spectra with NPG film and dense gold film, respectively. In contrast, the SPR sensor with an antibody-functionalized dense gold film could detect BaP at concentration as low as 100 pmol·L^−1^, the resonance-wavelength shift is 0.9 nm.

**Figure 8:**
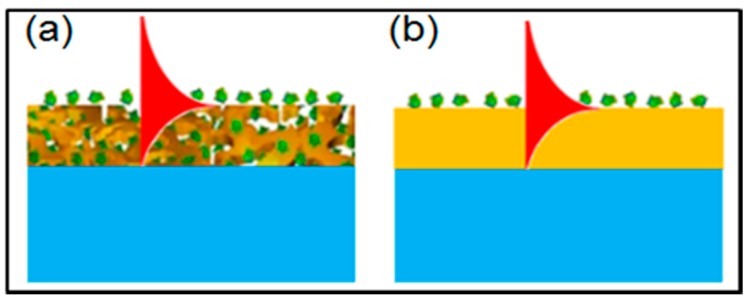
Schematic diagram showing the difference in evanescent wave interaction with adsorbed molecules between (**a**) the NPG film and (**b**) the dense gold film.

Figure 8 schematically shows the difference of the evanescent-wave interaction with the adsorbed analyte molecules between the NPG and dense gold films for understanding the high sensitivity of the NPG-based SPR. The dense gold film allows for formation of only a monolayer of adsorbed analyte molecules, thereby limiting the interaction depth to the monolayer thickness. The evanescent field inside the dense gold film cannot interaction with the analyte molecules. In contrast, the NPG film enables to adsorb a large amount of analyte molecules along the whole thickness of the film, leading to the spatial overlapping between the evanescent field and the adsorbed analyte molecules. Therefore, the interaction depth for the NPG-film-based SPR is as large as the the film thickness. It is the large extension of the interaction depth that makes the NPG-film-based SPR sensor much more sensitive than the conventional SPR sensors with dense gold film.

## 4. Conclusions

In this paper, a SPR sensor based on a NPG film prepared by a combination of sputtering and dealloying was fabricated for trace detection of BaP in water. The NPG films were modified with two surface chemistry approaches. The detection limit of the SPR sensor with the hydrophobic NPG film is 1 nmol·L^−1^, while the SPR sensor with the antibody-functionalized NPG film could detect BaP at concentrations as low as 5 pmol·L^−1^. Furthermore, the sensitivity of the NPG-based SPR sensor is about 20 times larger than that of the conventional SPR sensors with dense gold film. This could be contributed to the NPG film’s double roles of analyte enrichment and supporting a surface plasmon wave. The proposed NPG-based SPR sensor shows great potential for the trace detection of BaP in the environment.

## Figures and Tables

**Table 1:** Principal sensors employed in BaP detection.

Detection Principle	Detection Limit	Reference
Surface enhanced Raman spectroscopy (SERS)	5 nmol·L^−1^	[25]
Surface Plasmon Resonance (SPR)	0.2 nmol·L^−1^	[26]
Quartz crystal microbalance (QCM)	10 nmol·L^−1^	[29]
Immunoassay using electro- switchable biosurfaces	40 pmol·L^−1^	[28]
Electrochemistry (EC)	23.8 pmol·L^−1^	[27]
Fluorescence spectroscopy	11.9 pmol·L^−1^	[12]
NPG film based SPR	5 pmol·L^−1^	This work
